# How useful is the assessment of lymphatic vascular density in oral carcinoma prognosis?

**DOI:** 10.1186/1477-7819-5-140

**Published:** 2007-12-11

**Authors:** Adhemar Longatto Filho, Tiago Gil Oliveira, Céline Pinheiro, Marcos Brasilino de Carvalho, Otávio Alberto Curioni, Ana Maria da Cunha Mercante, Fernando C Schmitt, Gilka JF Gattás

**Affiliations:** 1 Life and Health Sciences Research Institute (ICVS), School of Health Sciences, University of Minho, Braga, Portugal; 2 Service of Head and Neck Surgery, Heliopolis Hospital, São Paulo, Brazil; 3 Department of Pathology, Heliopolis Hospital, São Paulo, Brazil; 4 Medical Faculty, Department of Pathology, University of Porto, Porto, Portugal; 5 IPATIMUP-Institute of Molecular Pathology and Immunology of University of Porto, Porto, Portugal; 6 Department of Legal Medicine, Ethics and Occupational Health, School of Medicine, Instituto Oscar Freire, São Paulo, Brazil

## Abstract

**Background:**

Lymphatic vessels are major routes for metastasis in head and neck squamous cell carcinoma (HNSCC), but lymphatic endothelial cells (LECs) are difficult to recognize in tumor histological sections. D2-40 stains podoplanin, a molecule expressed in LECs, however, the potential prognostic usefulness of this molecule is not completely understood in HNSCC. We aimed to investigate the value of assessing peritumoral and intratumoral lymphatic vessel density (LVD) as prognostic marker for HNSCC.

**Methods:**

Thirty-one cases of HNSCC were stained for D2-40 and CD31. LVD and blood vessel density (BVD) were assessed by counting positive reactions in 10 hotspot areas at ×200 magnification.

**Results:**

D2-40 was specific for lymphatic vessels and did not stain blood vascular endothelial cells. LECs showed more tortuous and disorganized structure in intratumoral lymphatic vessels than in peritumoral ones. No statistical differences were observed between peritumoral-LVD and intratumoral-LVD or between peritumoral-BVD and intratumoral-BVD. Tumor D2-40 staining was positively associated with lymphatic vessel invasion (*p *= 0.011).

**Conclusion:**

LVD is a powerful marker for HNSCC prognosis. We found significant differences in peritumoral and intratumoral D2-40 immunoreactivity, which could have important implications in future therapeutic strategies and outcome evaluation.

## Background

Most of oral cavity cancers are squamous cell carcinomas and, although they are accessible to biopsy and early identification, at the time of diagnosis, most of them have already metastasized. Metastatic spread to regional lymph nodes through the lymphatic system is one of the major pathways by which head and neck squamous cell carcinoma (HNSCC) disseminates. The mechanisms that tumors use to metastasize are well documented concerning the hematogenous spread, but lymphatic spread is not so well understood. However, recent findings show its importance in several human malignancies, including HNSCC [1].

The study of lymphangiogenesis, growth and production of new lymphatic vessels under several physiological and pathological conditions has gained more attention in recent years. Presently, there is no consensus whether the major pathway of lymphatic spread is through the development of lymphatic vessels intra or peritumorally, with studies published supporting each of the possibilities [2,3].

To understand the mechanisms underlying lymphangiogenesis it is essential to understand how lymph vessels develop and find specific markers for them. During embryonic development endothelial cells express lymphatic vascular endothelial receptor (LYVE-1) and vascular endothelial growth factor receptor (VEGFR-3) and, afterwards, the expression of the homeobox gene *Prox1 *commits these cells to the lymphatic lineage [4]. Members of VEGF family are closely related to the lymphatic vessels spread. Lymphangiogenesis largely depends on VEGFC signalling and the activity of the receptor VEGFR3 [5,6]. Another molecule involved in this process is Podoplanin, a transmembrane glycoprotein that may contribute to lymphatic endothelial cell adhesion and migration and to the formation of lymphatic connections [7].

In the last few years, specific lymphatic endothelial markers have been discovered, such as anti-LYVE-1 [8], anti-Prox-1 [9], anti-VEGFR-3, anti-podoplanin [2] and D2-40 [10], which are able to stain lymphatic endothelial cells (LECs). The recently discovered marker D2-40 was found to be highly specific for LECs, not staining blood vessel endothelial cells. Afterwards, it was also demonstrated that D2-40 and anti-podoplanin had similar expression of its antigens (M2A and podoplanin, respectively) in human developing testis, testicular carcinoma in situ and germ-cell tumors [11]. Evangelou compared both markers and showed that they had similar specificity staining for LECs [12] and, finally, Schacht et al reported that D2-40 recognized the same molecule as anti-podoplanin [13]. Presently, D2-40 is accepted as a marker for M2A antigen, also called podoplanin, and it is known that besides LECs, D2-40 is also expressed by other types of normal and neoplastic cells [10,14].

The aim of this study was to investigate the role of lymphatic vascular density (LVD) in a series of HNSCC and evaluate whether intra or peritumoral LVD correlated with the presence of lymph node invasion and distant metastasis, and with patients clinical outcome. For that purpose, D2-40 was used in due to of its higher specificity, as suggested by Van den Eyden *et al*. [15], making it possible to distinguish blood vascular density (BVD) from LVD, and study its role in HNSCC dissemination. Finally, D2-40 staining of tumor cells was also evaluated, in order to study the potential role of podoplanin in neoplasia development and invasion.

## Patients and methods

The material studied comprised formalin-fixed paraffin embedded tissue samples from 31 previously untreated patients with squamous cell carcinoma of the oral cavity. Tumor sites included: 4 of the retromolar area, 10 of the floor of mouth, 3 of the lower gingival area and 14 of the oral tongue. All patients received local resection and elective lymph node neck dissection (unilateral or bilateral) at Service of Head and Neck Surgery, Hospital Heliopolis, Sao Paulo – Brazil. None of these patients had received any drugs therapy before surgery. All patients had histologically confirmed squamous cell carcinoma. Fourteen patients received adjuvant postsurgical radiotherapy following the clinical protocol of the Hospital. These patients were submitted to resection margins, positive lymph nodes and/or perineural invasion. Additionally, all patients have had at least 24 months of clinical follow up. The group was constituted by patients with a median age of 50 years (range, 38 – 70 years) and included 29 men and 2 women. Of these, 29 had a prolonged smoking history and 26 had chronic alcohol consumption.

The distribution of patients was made according to the post-surgery TNM classification based on the American Joint Committee on Cancer (AJCC) and the Union Internationale Contre le Cancer (UICC) and the clinical classification was based on AJCC-UICC; additionally, the T, N, and M characteristics are combined to produce a "stage" of the cancer, from I to IVB [16].

### D2-40 Immunohistochemical procedure

Immunohistochemistry was carried out using the avidin-biotin-peroxidase complex assay, with the monoclonal antibody D2-40 (DAKO Corporation, Carpinteria, CA, USA). Briefly, deparaffinized and re-hydrated sections were immersed in 0.01 M citrate buffer (pH 6.0) and heated at 98°C for 20 min.; then slides were incubated with 0.3 % hydrogen peroxide in methanol for 30 min., followed by incubation with Normal Horse Serum (Vector Laboratories Inc., CA, USA) for 20 min., at room temperature, before incubating with the primary antibody diluted 1:100, overnight at 4°C. Sections were sequentially washed in PBS 1× and incubated with Biotinylated Universal Secondary antibody (Vector Laboratories Inc., CA, USA) for 30 min., Vectastain^® ^Elite ABC reagent (Vector Laboratories Inc., CA, USA) for 45 min. at 37°C, and developed with 3,3'-diamino-benzidine (DAKO Corporation, Carpinteria, CA, USA) for 10 min. Negative controls were performed by omitting the primary antibody and, as positive control, tonsil tissue was used.

### CD31 Immunohistochemical procedure

For CD31, the immunohistochemistry was carried out with the streptavidin-biotin-peroxidase complex technique (Ultravision Detection System Anti-polyvalent, HRP, Lab Vision Corporation, Fremont, CA, USA), using specific primary antibody raised against CD31/PECAM-1 (rabbit monoclonal antibody, clone Ab-1/JC/70A, Neomarkers, Freemont, CA, USA) diluted 1:50. Briefly, deparaffinized and rehydrated sections were heated up to 98°C for 20 min. in 0.01 M citrate buffer (pH 6.0). Endogenous peroxidases were inactivated with 3 % hydrogen peroxide in methanol for 10 min., followed by washing in PBS/Tween. Tissue sections were incubated with blocking solution for 10 min. and incubated with the primary antibody for 60 min. at room temperature. Sections were then sequentially washed in PBS/Tween and incubated with biotinylated goat anti-polyvalent antibody for 10 min., streptavidin peroxidase for 10 min., and developed with 3,3'-diamino-benzidine (DAB Substrate System; Lab Vision Corporation, Fremont, CA) for 10 min. Appropriated positive and negative controls were included in each run. Negative controls were performed by omission of the primary antibody and angiosarcoma was used as positive control. The slides were counterstained with haematoxylin and mounted with Synthetic Mountant Entellan (Merck, Darmstadt, Germany).

### Immunohistochemical evaluation

The immunohistochemical positive reaction of D2-40 and CD31 antibodies was evaluated considering its expression in the cytoplasm of lymphatic and blood endothelial cells, respectively. The evaluation was performed blindly and both LVD and BVD were assessed as postulated by Weidner *et al *[17], with slightly modifications. Microvessel was defined as a single endothelial cell or a cluster of endothelial cells positive for D2-40 or CD31, respectively, sitting around a visible lumen clearly separate from adjacent microvessels and from other connective tissue components. Additionally, as lymphatic vessels could generally appear as distorted and overlapped structures in cancer setting, the packed vessels were assumed as one lymphatic unit. In contrary, blood vessels commonly do not display distorted and packaged appearance. The number of vessels was quantified at ×200 (×20 objective lens and ×10 ocular lens) magnification. A median of 10 hot spot fields was defined as vessel density. The examination of each hotspot corresponds to a number of vessels confined to an area of 0.15 mm^2^. Both D2-40 and CD31 immunohistochemical positive reactions were independently counted in lymphatic and blood vessels from intratumoral and peritumoral areas. Intratumoral area was defined as the stromal tissue within two or more neoplastic aggregates, and peritumoral area was defined as the stroma tissue surrounding these neoplastic mass. D2-40 and CD31 positivity in tumor cells was classified, considering immunoreaction extension as negative (negative or weak immunoreaction) and positive (moderate to strong immunoreaction). For evaluation of lymphatic and blood vessels invasion, only D2-40 and CD31 positive vessels occupied by neoplastic cells, respectively, were considered. Seven cases were excluded for CD31 evaluation due to technical limitations.

### Statistical analysis

Data were stored and analyzed using the SPSS statistical software (for Windows, version 14.0, Chicago, IL). The Shapiro-Wilk test was applied to assess normality of the results. Data was examined for statistical significance using the T-Student, the One-Way ANOVA, the Mann-Whitney U, the Kruskal-Wallis and the Pearson's chi-square (χ^2^) tests, as appropriate, being threshold for significance *p *values < 0.05.

## Results

**Figure 1:**
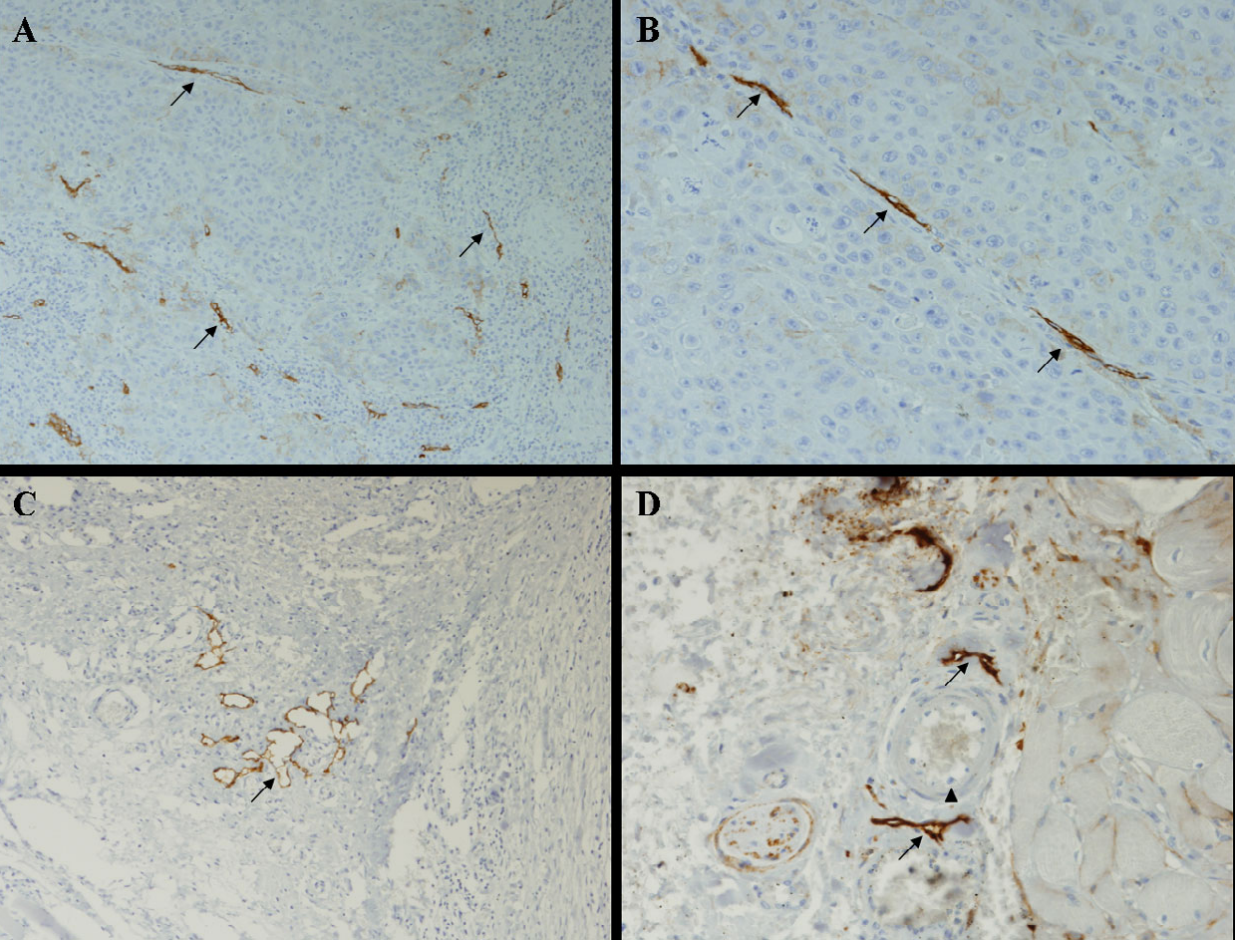
Lymphatic vessels (black arrows) were specifically stained with D2-40, an antibody against podoplanin (A, B, C, D), that did not stain blood vessels (arrow head). A (×100), B (×200), C (×100), D (×200).

**Figure 2:**
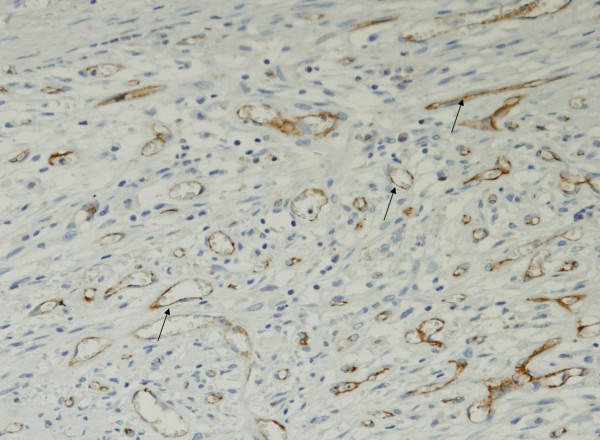
All samples evaluated were found to have positive reaction to CD31, staining vascular endothelial cells (black arrows). (×200).

The comparison of vascular densities between peritumoral and intratumoral areas assessed by the lymphatic marker D2-40 and blood vessels marker CD31 did not show significant differences. Peritumoral-LVD and intratumoral-LVD medians were 6.8 and 6.2 vessels in 10 hotspots, respectively (*p *= 0.488); and peritumoral-BVD and intratumoral-BVD were 9.3 and 7.2 vessels per 10 hotspots, respectively (*p *= 0.299). Figures 1 and 2 show representative immunoreactions for D2-40 and CD31, respectively.

Table 1 depicts the significance of LVD and BVD related with clinical -pathological data. Interestingly, BVD did not correlate with classical parameters of tumor aggressiveness.

**Table 1:** Associations of LVD and BVD with the clinic-pathological data.

	Peritumoral LVD*			**Intratumoral LVD**^¥^			Peritumoral BVD*			Intratumoral BVD*		
	**n**	**Median**	***p***	**n**	**Mean**	***p***	**n**	**Median**	***p***	**n**	**Median**	***p***
**Age**			1.000			0.569			0.590			0.266
**Gender**			0.560			0.743			----			----
**Smoking history**			0.533			0.295			0.532			0.250
**Alcohol consumption**			0.867			0.502			0.773			0.896
**Mouth region**			0.454			0.118			0.845			0.495
Retromolar	**4**	6.2		**4**	7.8		**4**	8.1		**4**	4.6	
Oral floor	**10**	5.2		**10**	5.3		**8**	8.0		**7**	8.3	
Lower gingiva	**3**	7.1		**3**	7.9		**3**	10.2		**3**	15.9	
Tongue	**13**	7.5		**14**	6.4		**11**	9.7		**10**	7.4	
**Histological Differentiation**			0.629			0.526			0.987			0.711
**Desmoplasia**			0.986			0.869			0.288			0.085
**Perineural infiltration**			0.780			0.721			0.833			0.538
**T**			0.491			0.443			1.000			0.567
pT1 + pT2	**11**	7.0		**12**	6.0		**9**	8.7		**7**	6.9	
pT3 + pT4	**19**	6.7		**19**	6.6		**17**	8.7		**17**	7.6	
**N**			0.722			0.767			**0.085**			0.202
pN0 + pN1	**17**	7.0		**17**	6.3		**14**	6.7		**13**	6.9	
pN2 + pN3	**13**	6.7		**14**	6.5		**12**	9.6		**11**	7.6	
**Clinical Staging**			0.423			0.998			0.652			0.819
**Metastasis**			0.717			0.159			0.355			0.698
Absent	**25**	6.5		**26**	6.6		**22**	9.0		**20**	7.4	
Present	**5**	7.1		**5**	5.1		**4**	6.5		**4**	6.0	
**Local recurrence**			**0.052**			0.618			0.544			0.874
Negative	**22**	6.5		**22**	6.3		**19**	8.4		**17**	7.5	
Positive	**8**	8.4		**9**	6.7		**7**	9.7		**7**	7.2	
**Outcome**			**0.083**			0.859			0.314			**0.052**
Disease free	**14**	6.0		**14**	6.2		**11**	6.9		**10**	7.2	
Death for other reasons	**3**	8.7		**3**	6.6		**3**	10.2		**3**	15.9	
Death by cancer	**13**	7.8		**14**	6.6		**12**	9.0		**11**	5.8	

* Mann-Whitney U test and Kruskal-Wallis test when k > 2; ^¥ ^T-Student test and One-Way ANOVA when k > 2)

**Figure 3:**
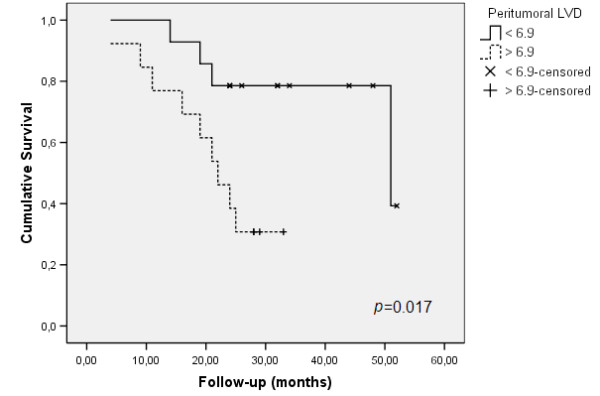
Survival rates related with peritumoral LVD.

Remarkably, peritumoral LVD was correlated with a shorter survival (*p *= 0.017; Figure 3).

**Figure 4:**
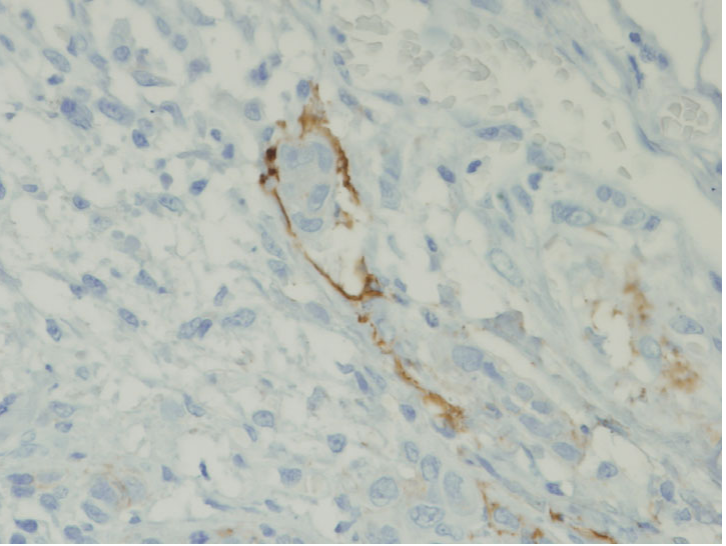
Invasion of lymphatic vessels was found, with tumor cells inside the lumen of the lymphatics. (×200).

Lymphatic invasion (Figure 4) was observed in 25 cases with a statistical tendency to be associated with both peri and intratumoral-LVD (*p *= 0.079 and *p *= 0.078, respectively). Table 2 shows the correlation of both lymphatic and blood vessel invasion with the clinical-pathological parameters.

**Table 2:** Association of lymphatic and blood vessels invasion, as well as positive D2-40 immunoreaction in tumor cells, with the clinic-pathological data.

		**Lymphatic invasion**		**Lymphatic invasion > 3**		**Blood vessel invasion**			**D2-40 positivity in tumor cells**		
	**n**	**%**	***p***	**%**	***p***	**n**	**%**	***p***	**n**	**%**	***p***
**Age**			**0.056**		0.886			0.157			0.474
> 50	**15**	66.7		40.0		**13**	0.0		**15**	80.0	
≤ 50	**16**	93.8		37.5		**14**	14.3		**16**	68.8	
**Gender**			0.474		**0.066**			0.773			0.419
Female	**2**	100.0		100.0		**1**	0.0		**2**	50.0	
Male	**29**	79.3		34.5		**26**	7.7		**29**	75.9	
**Smoking history**			0.257		0.245			0.678			0.419
**Alcohol consumption**			0.232		**0.038**			0.603			0.746
No	**5**	100.0		80.0		**3**	0.0		**5**	80.0	
Yes	**26**	76.9		30.8		**24**	8.3		**26**	73.1	
**Mouth region**			0.472		0.317			0.853			0.653
**Differentiation**			0.111		0.968			**0.077**			0.982
Well differentiated	**7**	57.1		42.9		**6**	0.0		**7**	71.4	
Moderate differentiated	**16**	93.8		37.5		**13**	0.0		**16**	75.0	
Poor differentiated	**8**	75.0		37.5		**8**	25.0		**8**	75.0	
**Desmoplasia**			0.724		0.909			0.313			0.867
**Perineural infiltration**			0.283		**0.035**			0.693			0.319
Negative	**20**	75.0		25.0		**17**	5.9		**20**	80.0	
Positive	**11**	90.9		63.6		**10**	10.0		**11**	63.6	
**T**			0.763		0.625			0.299			0.935
**N**			0.791		0.756			0.189			0.253
**Clinical Staging**			0.998		0.640			0.467			0.294
**Metastasis**			0.232		0.286			0.540			0.746
**Tumor recurrence**			0.457		0.675			**0.013**			0.540
Negative	**22**	77.3		36.4		**20**	0.0		**22**	77.3	
Positive	**9**	88.9		44.4		**7**	28.6		**9**	66.7	
**Outcome**			0.290		**0.039**			0.259			0.867
Disease free	**14**	71.4		14.3		**12**	0.0		**14**	78.6	
Dead for other reasons	**3**	66.7		66.7		**3**	0.0		**3**	66.7	
Dead by the cancer consequences	**14**	92.9		57.1		**12**	16.7		**14**	71.4	

Lymphatic invasion = any number of invaded lymphatic vessel. Lymphatic invasion > 3 = this number represents a median value.

**Figure 5:**
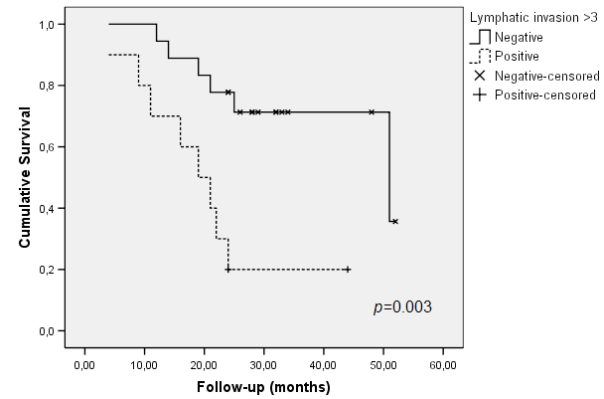
Survival rates related with D2-40 positive invaded vessels.

Perineural infiltration correlated with lymphatic invasion superior than three vessels per case (*p *= 0.035) and worse outcome was significantly more observed also in cases with prominent lymphatic invasion (*p *= 0.039). Importantly, lymphatic invasion observed in more than 3 vessels was associated with shorter survival (*p *= 0.003; Figure 5). This value was obtained with the median calculation for lymphatic vessel invasion.

Poorly differentiated tumors present a tendency to invade blood vessels (*p *= 0.077). Also, tumor recurrence was more frequent in cases with blood vessel invasion (*p *= 0.013). No association between blood vessel invasion and BVD was observed.

**Figure 6:**
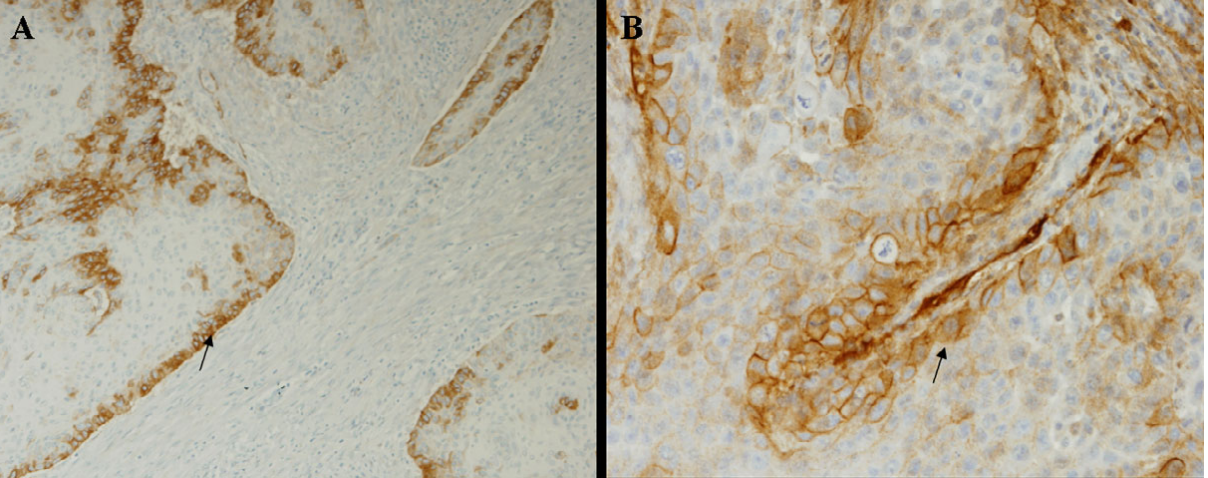
Some of the samples evaluated had malignant cells (black arrows) positively stained for D2-40 (A, B). A (×100), B (×200).

Some D2-40 immunoreaction was observed in epithelial tumor cells (Figure 6), however, no statistical correlation was observed between positive reactions of D2-40 in tumors cells and the clinical-pathological parameters (Table 2). D2-40 staining was positively associated with lymphatic vessel invasion; 84.0 % of the cases presenting lymphatic vessel invasion were positive for D2-40 expression in tumor cells while only 33.3 % of the negative cases for lymphatic vessel invasion presented D2-40 expression in tumor cells (*p *= 0.011).

## Discussion

Nodal metastatic invasion is believed to be one of the major predictors of HNSCC outcome [1,2]. It is still under discussion whether lymphatic dissemination is through lymphangiogenesis or through pre-existing lymphatics. It has been shown, however, that the structure of lymphatic vessels differs in these types of tumors, as they had a higher number of lymphatic vessels, observed both intratumorally and peritumorally [3]. In our study we observed that lymphatic structure intratumorally was disorganized, with frequently tortuous or collapsed lumen and that lymphatic invasion was present in most of the samples. Together with the fact that HNSCC metastasize frequently to lymph nodes, these findings support the hypothesis that tumor lymphangiogenesis may be a fundamental pathway for neoplasia dissemination in HNSCC [2].

Nevertheless, there is still some controversy whether the major contributors are intratumoral or peritumoral lymphatic vessels. Franchi *et al*. [3] suggest that probably both of them have a role in dissemination, but the relevance of each one must be further assessed. In their study they show a correlation between LVD peritumorally and lymph node involvement. On the contrary, Kysas *et al*. [2] show a correlation between LVD intratumorally and lymph node involvement. It was observed that intratumoral LVD is composed by disorganized lymphatic vessels which seriously limits the usefulness for an effective conduit to metastization, comparing with the fully functional peritumorally lymphatic vessels. Importantly, in our series we observed that peritumoral LVD, but not intratumoral LVD, was significantly associated with recurrence, which could reinforce the hypothesis that peritumoral lymphatic vessels are competent to participate in the neoplastic escape. We also observed that cases where the tumor cells invaded more than three lymphatic vessels strongly correlated with perineural invasion and poor clinical outcome. This is important because perineural invasion is a phenomenon that may affect the therapeutic decision making in dealing with head and a neck cancer and its occurrence is believed to be a remarkable sign of tumor aggressiveness [18].

Because D2-40 is being used mainly as a lymphatic vessel marker, staining of other cells rather than LECs is important to be distinguished to minimize errors in quantification of LVD. Besides that, it could also give new clues to understand the functions and importance of podoplanin. Franchi *et al*. reported no staining of tumor cells in their HNSCC samples [3]. On the contrary, we found that D2-40 stained the majority of the tumor cells (n = 23 or 74%) in HNSCC samples evaluated in this study. Concordantly with our observation, there is also data showing that D2-40 actually stains carcinomas and other malignancies [10,13,14]. It was reported that D2-40 is specific for M2A region of podoplanin, a molecule found to be overexpressed in some tumors cells [13]. These data together with the knowledge about the role of podoplanin in cytoskeleton reorganization, leads us to believe that podoplanin might play a role in neoplastic development, as previously proposed by Schacht *et al*. [13]. Supporting this hypothesis, Wicki *et al*. [19] showed, using an experimental mice model, that overexpression of podoplanin induces cellular alterations resulting in increased cellular migration. In accordance to our results, they also observed a peripheric tumoral staining pattern. Based on these findings, they suggested that podoplanin might in fact play a pivotal role in a novel molecular pathway of collective cell migration independently from other processes such as cadherin switch and epithelial-mesenchymal transition [19]. Dumoff and colleagues [20] recently reported that low immunoreactivity for D2-40 in tumor cells correlates with lymphatic invasion and nodal metastasis in squamous cell carcinoma of the uterine cervix. In spite of these evidences, we did not find significant differences in D2-40 positive reaction in tumor cells and the clinical parameters of cancer prognosis. Against to some evidences documented in cervical cancer [21], we found that D2-40 positive reaction in tumor cells correlates with lymphatic invasion, but not with lymph node invasion, corroborating just in part the findings recently reported by Yuan and co-workers [22]. These authors suggested that podoplanin may play a role in promoting the spread of tumor cells through lymphatic vessels because podoplanin-expressing tumor cells were also observed inside lymphatic vessels; additionally, they also observed the tumor cells expressing high levels of podoplanin even in regional lymph nodes. Presently, these interesting findings were not corroborated by our observations. We actually observed that positive D2-40 decorating malignant cells and LVD highlighted by D2-40 staining both are associated to lymphatic invasion. Nevertheless, the significance of these contradictory observations is not currently recognized. However, two motivating studies have demonstrated that lymphatic markers were differentially expressed by LECs from different organs, which indicate a clear phenotypic heterogeneity [23]; and that lymphangiogenesis in oral SCC vary depending on the region within the tumor tissue [24]. Accordingly, we can hypothesize that tumor cells of same histological pattern could also differently express podoplanin according to the organ where the neoplasia grows.

## Conclusion

our results demonstrate that D2-40 can be considered a useful marker to predict HNSCC aggressiveness because peritumoral LVD and D2-40 positive reaction in cancer cells, both highlighted with D2-40 positive staining, were strongly associated with lymphatic vessel invasion and clinical parameters of worse prognosis.

## Competing interests

The author(s) declare that they have no competing interests.

## Authors' contributions

ALF: Chief author. Created the idea, co-author selection. Participated in the design of the study, and drafted the manuscript.

TGO: Carried out immunohistochemistry, and photomicrography, revised the manuscript draft.

CP: Participated in the laboratory design of the study and performed the statistical analysis (cooperating with a specialist statistician).

MBC and OAC: Performed clinical and surgical work.

AMM: Performed the surgical pathology diagnosis.

FCS: Performed D2-40 evaluation and revision of the manuscript.

GJG: Approved study design, participated in the sequence alignment, and revised references and final manuscript.

All authors read and approved the final manuscript.
